# Diagnostic Utility of the Carbohydrate Antigen 19-9 (CA 19-9)/C-reactive Protein (CRP) Ratio for Predicting Malignancy in Obstructive Jaundice

**DOI:** 10.7759/cureus.118019

**Published:** 2026-10-16

**Authors:** Nur Alam Mohim, Murshidul Arefin, Md. Kawsar Bhuiyan, Anupam Debnath, Golam Mahmud Rayhan, Amit Chowdhury, Muhammad Salauddin, Halima Khatun, Shah Md. Ezaz-Ul Haque, Mohammad Saief Uddin

**Affiliations:** 1 Department of Surgery, Rupganj Upzilla Health Complex, Dhaka, BGD; 2 Department of Hepatobiliary, Pancreatic, and Liver Transplant Surgery, Bangladesh Medical University, Dhaka, BGD; 3 Department of Hepatobiliary Surgery, Evercare Hospital, Dhaka, BGD; 4 Department of Surgery, National Gastroliver Institute and Hospital, Dhaka, BGD; 5 Department of Hepatobiliary Surgery, United Medical College Hospital, Dhaka, BGD; 6 Department of Dermatology, Directorate General of Health Services, Dhaka, BGD; 7 Department of Public Health, Dhaka Medical College, Dhaka, BGD

**Keywords:** biliary obstruction, biomarker ratio, ca 19-9, c-reactive protein, malignancy, obstructive jaundice

## Abstract

Background: Differentiating malignant from benign obstructive jaundice remains difficult, particularly when conventional tumour markers are elevated by cholestasis or superimposed inflammation. Carbohydrate antigen 19-9 (CA 19-9) is widely used in pancreatobiliary malignancy, but its value is reduced in benign biliary obstruction. C-reactive protein (CRP), an inflammatory marker, may help contextualise CA 19-9 elevation.

Objective: The primary objective was to determine the diagnostic discrimination of the CA 19-9/CRP ratio for malignancy among patients with obstructive jaundice. Secondary objectives were to compare its observed performance with that of CA 19-9 alone and the CA 19-9/bilirubin ratio, and to estimate an exploratory threshold with corresponding sensitivity and specificity.

Methodology: This comparative cross-sectional study was conducted in the Department of Hepatobiliary, Pancreatic and Liver Transplant Surgery, Bangladesh Medical University, Dhaka, over one year. Ninety adult patients with obstructive jaundice were enrolled by purposive sampling: 45 with malignant obstruction and 45 with benign obstruction after definitive diagnosis by imaging, operative findings, and histopathology where applicable. Serum CA 19-9, CRP, and bilirubin were measured in all participants. Group comparisons were performed using the chi-square test, Fisher’s exact test, Student’s t-test, or Mann-Whitney U test as appropriate. Receiver operating characteristic analysis was used to determine discriminatory performance.

Results: Patients with malignant obstruction were older than those with benign disease (57.42 ± 8.4 vs. 44.82 ± 16.18 years, P = 0.001). Median CA 19-9 was higher in malignant than in benign obstruction (370 vs. 41.4 U/mL; P = 0.004). Median CRP was lower in malignant than in benign obstruction (15.2 vs. 88.0 mg/L; P = 0.001). The CA 19-9/CRP ratio was higher in malignant than in benign obstruction (42.92 vs. 0.70; P = 0.001). On receiver operating characteristic analysis, the CA 19-9/CRP ratio had the strongest performance for predicting malignancy, with an area under the curve of 0.812, a sensitivity of 84.4%, a specificity of 71.1% and an optimal cut-off of 7.23. This exceeded the performance of CA 19-9 alone (area under the curve 0.677) and the CA 19-9/bilirubin ratio (area under the curve 0.619).

Conclusions: The CA 19-9/CRP ratio demonstrated better discriminative ability than CA 19-9 alone for identifying malignant obstructive jaundice in the study participants. This inexpensive and readily available ratio may be useful as an adjunctive preoperative marker, especially where access to advanced diagnostic modalities is limited.

## Introduction

Obstructive jaundice is a common clinical syndrome caused by blockage of bile flow and remains a major diagnostic challenge, as the underlying pathology may be benign or malignant, with similar biochemical and imaging patterns [1-3]. Early differentiation is important because delayed diagnosis of malignancy can postpone definitive treatment, whereas overdiagnosis of malignancy may expose patients with benign obstruction to unnecessary invasive investigations [4,5]. The most widely used serum biomarker in pancreaticobiliary disease is carbohydrate antigen 19-9 (CA 19-9) [6]. It is a mucin-bound glycoprotein secreted by pancreatic and biliary ductal epithelium and has long been used in the assessment of pancreatic and biliary tract malignancies [7,8]. CA 19-9 is widely used in the evaluation of pancreatic cancer and cholangiocarcinoma. However, its diagnostic specificity is limited because levels may also rise in benign biliary obstruction and inflammatory hepatobiliary conditions [9-11]. A major problem is that CA 19-9 levels may be elevated in various benign conditions, including obstructive jaundice, cholangitis, pancreatitis and other inflammatory or systemic diseases. False-positive elevations have been reported in benign biliary obstruction, Mirizzi syndrome, autoimmune and chronic inflammatory hepatobiliary disease, respiratory disease and even heavy tea consumption [12-15]. Similar concerns have also been raised in cholangiocarcinoma work-ups and other gastrointestinal malignancies, where CA 19-9 alone may be misleading [16,17]. Researchers have therefore attempted to enhance the clinical utility of CA 19-9 by normalising it to bilirubin or inflammatory indicators [18,19]. C-reactive protein (CRP) is a non-specific acute-phase reactant associated with infection, tissue injury and neoplastic inflammation, and persistent CRP elevation has also been associated with poorer oncological outcomes in other cancers [20]. Earlier studies have indicated that biomarker ratios may help distinguish benign from malignant obstruction in cohorts of patients with obstructive jaundice. These findings suggest that the CA 19-9/CRP ratio might be superior to either CA 19-9 alone or the CA 19-9/bilirubin ratio in selected populations, although it has not been externally validated [18,21]. As the diagnostic pathway still involves imaging, serum markers should be interpreted as adjuncts rather than substitutes; however, they may be particularly helpful when advanced diagnostic assessment is unavailable or when early triage decisions are required [5,22,23]. Although previous studies have investigated CA 19-9-based ratios, diagnostic estimates and proposed thresholds may vary according to the disease spectrum and clinical setting. This study assessed the CA 19-9/CRP ratio alongside CA 19-9 alone and the CA 19-9/bilirubin ratio in a single-centre cohort in Bangladesh. Thus, our study aimed to provide exploratory, setting-specific comparative data to assess the diagnostic utility of the CA 19-9/CRP ratio for predicting malignancy in obstructive jaundice, rather than to establish a new universal cut-off.

## Materials and methods

Study design and setting

This comparative cross-sectional study was conducted in the Department of Hepatobiliary, Pancreatic and Liver Transplant Surgery, Bangladesh Medical University, Dhaka, Bangladesh (formerly Bangabandhu Sheikh Mujib Medical University), from January 2024 to December 2024.

For this narrative literature overview, we searched PubMed, Google Scholar and Scopus for reports published between 2010 and 2025. Search terms included combinations of "obstructive jaundice", "CA 19-9", "carbohydrate antigen 19-9", "C-reactive protein", "CRP", "bilirubin", "biomarker ratio", "benign", "malignant" and "diagnostic accuracy". We prioritised human clinical studies relevant to biliary obstruction. This overview was not designed as a systematic review or meta-analysis.

Participants

Adult patients admitted with obstructive jaundice were screened. Obstructive jaundice was determined clinically and biochemically, with a serum total bilirubin level exceeding 2.0 mg/dL and supportive imaging showing biliary obstruction. Patients who had previously undergone biliary drainage, those with prehepatic or hepatic jaundice and those with disseminated malignant disease were excluded. Purposive sampling was used to enrol 45 patients with malignant obstructive jaundice and 45 with benign obstructive jaundice after definitive diagnosis. Buderer's formula was used for sample size calculation.

Data collection and laboratory methods

Each subject underwent a thorough clinical examination and routine laboratory tests, and serum total bilirubin, CA 19-9 and CRP levels were determined. Venous blood samples were collected in the Department of Biochemistry. The Alinity C total bilirubin assay was used to measure total bilirubin. CA 19-9 was measured using chemiluminescent microparticle immunoassay (CMIA), and CRP was measured using a latex immunoassay. The conventional upper reference limit for CA 19-9 was 37 U/mL, and the normal CRP limit was below 5 mg/L.

Reference diagnosis

The final diagnosis of benign or malignant obstructive jaundice was established through instrumental investigations, operative findings and histopathology, where indicated. Ultrasonography, computed tomography, magnetic resonance imaging and endoscopic retrograde cholangiopancreatography were used as required for the diagnostic work-up.

Variables and outcomes

The principal index was the CA 19-9/CRP ratio. Other variables included age, gender, serum bilirubin, CA 19-9, CRP, diagnosis and management approach. The primary outcome was the differentiation between benign and malignant obstructive jaundice.

Statistical analysis

The data were entered into Microsoft Excel spreadsheets and analysed with SPSS version 23 (IBM Corp., Armonk, NY). Frequencies and percentages were calculated to represent categorical variables. Mean ± standard deviation or median with interquartile range was used to describe continuous variables, depending on the distribution. The chi-square test, Fisher's exact test or the Mann-Whitney U test was used to test group differences, as appropriate. Receiver operating characteristic curves were plotted to approximate the cut-off values, sensitivity, specificity, area under the curve and 95% confidence intervals. A P value of less than 0.05 was considered statistically significant.

Ethical considerations

The institutional review board of Bangladesh Medical University, Dhaka, Bangladesh (formerly Bangabandhu Sheikh Mujib Medical University), provided ethical clearance (Memo no. BSMMU/2023/4576; dated 30 November 2023). Written informed consent was obtained prior to data collection, and confidentiality was maintained during the study.

## Results

The results of the study on 90 patients with obstructive jaundice were analysed: 45 patients with malignant obstruction and 45 with benign obstruction. Periampullary carcinoma (18/45, 40%), gallbladder carcinoma (9/45, 20%) and pancreatic head carcinoma (8/45, 17.8%) were the predominant malignant aetiologies. Postoperative biliary stricture (12/45, 26.7%), choledocholithiasis (10/45, 22.2%) and hepaticolithiasis (10/45, 22.2%) were most prevalent among benign obstructions (Table 1).

**Table 1:** Distribution of study patients according to final diagnosis (n = 90). Data are presented as numbers and percentages within the malignant and benign groups.

Diagnosis	Frequency (*n*)	Percentage (%)
Malignant, *n* (%)		
Periampullary carcinoma	18	40.0
Carcinoma gall bladder	9	20.0
Carcinoma of the head of the pancreas	8	17.8
Klatskin tumour	6	13.3
Distal cholangiocarcinoma	4	8.9
Benign, *n *(%)		
Biliary stricture following bile duct injury	12	26.7
Choledocholithiasis	10	22.2
Hepaticolithiasis	10	22.2
Choledochal cyst	5	11.1
Mirizzi syndrome	4	8.9
Biliary pancreatitis	3	6.7
Benign biliary stricture other than bile duct injury	1	2.2

Patients with malignant obstruction were significantly older than those with benign obstruction (57.42 ± 8.4 vs. 44.82 ± 16.18 years, *P* = 0.001). Sex distribution also differed significantly (*P* = 0.029), with a female predominance in the benign group (73.3%) and a near-equal sex distribution in the malignant group (Table 2).

**Table 2:** Demographic profile of malignant and benign obstructive jaundice (N = 90). *P* values were calculated using the t-test and chi-square test, as applicable. A *P*-value < 0.05 was considered statistically significant. SD, standard deviation

Variable	Malignant (*n* = 45)	Benign (*n* = 45)	Test statistics	*P*-value
Age, mean ± SD (years)	57.42 ± 8.4	44.82 ± 16.18	*t* = 4.638 (df = 88)	0.001
Age range, years	35-72	18-70		-
Gender				
Male, *n* (%)	22 (48.9)	12 (26.7)	χ² = 3.829 (df = 1)	0.029
Female, *n* (%)	23 (51.1)	33 (73.3)		

Among patients with malignant aetiology, Whipple’s procedure was the most frequently performed intervention (21/45, 46.8%), followed by ERCP with stenting (11/45, 24.5%) and percutaneous transhepatic biliary drainage (5/45, 11.1%). In contrast, benign cases were more commonly managed with excision of the extrahepatic biliary tract with Roux-en-Y hepaticojejunostomy (11/45, 24.6%) and left lateral segmentectomy with EHBT excision and Roux-en-Y hepaticojejunostomy (9/45, 20.0%). ERCP with stenting was also performed in 9/45 (20.0%) patients with benign aetiology (Table 3).

**Table 3:** Management strategies according to benign and malignant aetiology (N = 90). EHBT, extrahepatic biliary tract; ERCP, endoscopic retrograde cholangiopancreatography; HJ, hepaticojejunostomy; PTBD, percutaneous transhepatic biliary drainage; Roux-en-Y HJ, Roux-en-Y hepaticojejunostomy

Management	Malignant (*n *= 45), *n* (%)	Benign (*n *= 45), *n* (%)
Whipple's procedure	21 (46.8)	0 (0.0)
Triple bypass	2 (4.4)	0 (0.0)
Extended cholecystectomy	2 (4.4)	0 (0.0)
Left lateral segmentectomy with EHBT excision and Roux-en-Y HJ	0 (0.0)	9 (20.0)
Hepatic bisectionectomy with gallbladder mass excision	1 (2.2)	0 (0.0)
Excision of EHBT with Roux-en-Y HJ	1 (2.2)	11 (24.6)
Taj Mahal hepatic resection and reconstruction by Roux-en-Y HJ	0 (0.0)	1 (2.2)
Cholecystectomy with choledocholithotomy	0 (0.0)	5 (11.1)
Choledocholithotomy with T-tube insertion	0 (0.0)	4 (8.9)
Open cholecystectomy	0 (0.0)	2 (4.4)
ERCP and stenting	11 (24.5)	9 (20.0)
Percutaneous transhepatic biliary drainage	5 (11.1)	1 (2.2)
Conservative management	0 (0.0)	2 (4.4)
Refused any procedure	1 (2.2)	1 (2.2)
Death	1 (2.2)	0 (0.0)

Tumour marker profiles differed markedly between groups. CA 19-9 levels were significantly higher in the malignant group (*P* = 0.004), whereas CRP levels were considerably higher in the benign group (*P* = 0.001). This divergence led to a significantly higher CA 19-9/CRP ratio in malignant obstruction (*P* = 0.001) (Table 4).

**Table 4:** Comparison of CA 19-9, serum bilirubin, CRP and CA 19-9/CRP ratio between study groups (N = 90). *P* values were calculated using the Mann-Whitney U test, where applicable. A *P*-value < 0.05 was considered statistically significant. CA 19-9, carbohydrate antigen 19-9; CRP, C-reactive protein; IQR, interquartile range

Biochemical marker	Malignant (*n* = 45)	Benign (*n* = 45)	Test statistic	*P*-value
CA 19-9, median (IQR), U/mL	370 (42.3-1001)	41.4 (23.6-168.9)	*U* = 1371.0	0.004
S. Bilirubin, median (IQR), mg/dL	11.5 (6.4-15.2)	4.5 (3-10.5)	*U* = 1424.0	0.004
CRP, median (IQR), mg/L	15.2 (8.27-24)	88.0 (34.5-130)	*U* = 294.5	0.001
CA 19-9/CRP ratio, median (IQR)	42.92 (3.38-126)	0.7 (0.26-3.08)	*U* = 1644.0	0.001

Receiver operating characteristic (ROC) analysis showed that the CA 19-9/CRP ratio had the highest discriminatory performance among the evaluated markers (Figure 1).

**Figure 1:**
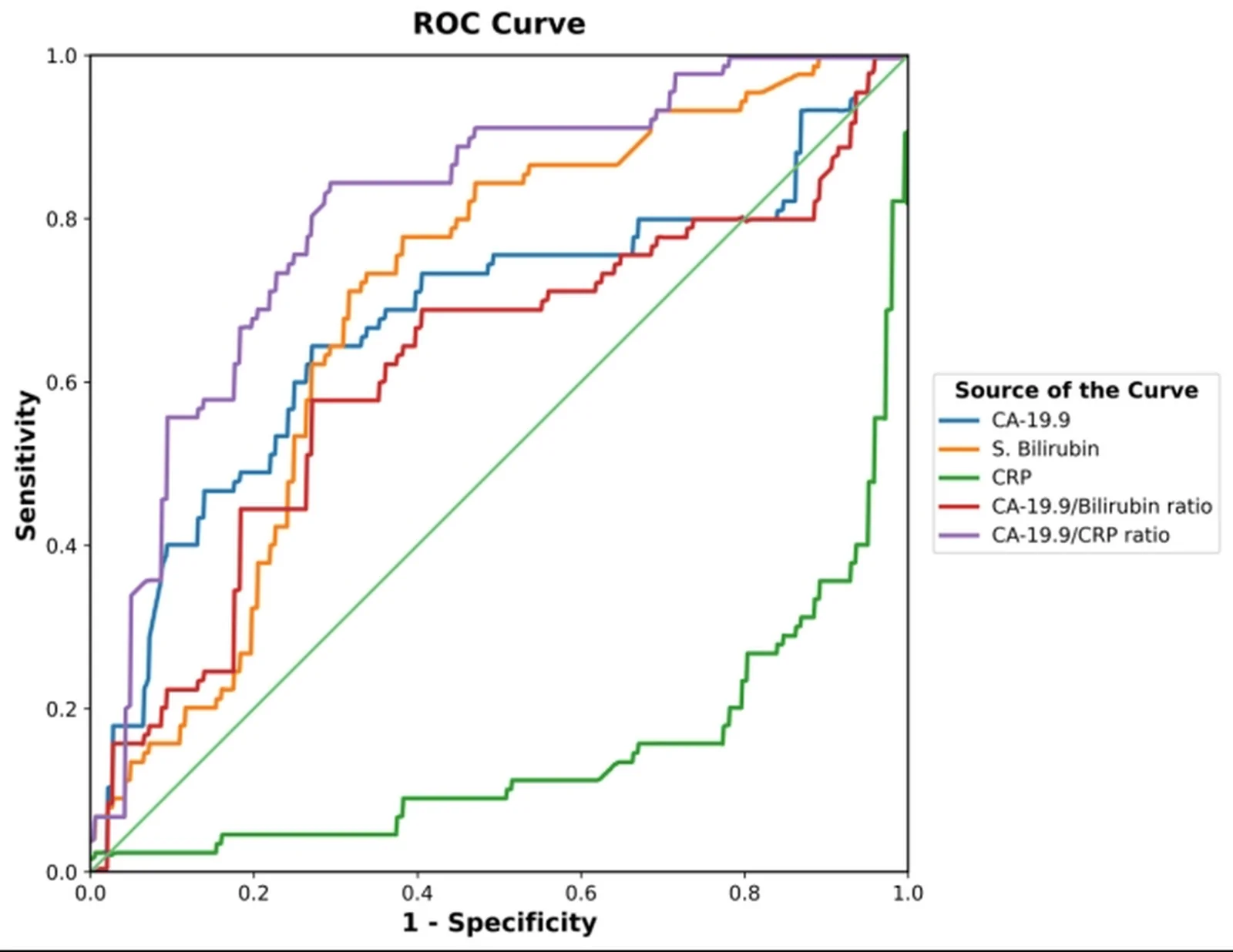
Receiver operating characteristic (ROC) curves of different biomarkers for predicting malignancy in obstructive jaundice. CA 19-9, carbohydrate antigen 19-9; S. bilirubin, serum bilirubin; CRP, C-reactive protein

The ratio achieved an area under the curve of 0.812, a sensitivity of 84.4%, a specificity of 71.1% and an optimal cut-off of 7.23 (Table 5).

**Table 5:** Receiver operating characteristic performance of candidate biomarkers for predicting malignancy (N = 90). CRP was higher in benign cases, so its AUC was below 0.5 and it was not useful on its own. CA 19-9, carbohydrate antigen 19-9; CRP, C-reactive protein; AUC, area under the curve; CI, confidence interval

Marker	Cut-off	Sensitivity (%)	Specificity (%)	AUC	*P*-value	95% CI
CA 19-9 (U/mL)	103.5	64.4	66.7	0.677	0.004	0.563-0.792
Serum bilirubin (mg/dL)	7.32	68.9	68.9	0.703	0.001	0.593-0.814
CRP (mg/L)	35.86	26.7	20.0	0.145	0.001	0.064-0.227
CA 19-9/bilirubin ratio	28.46	57.8	73.3	0.619	0.053	0.500-0.737
CA 19-9/CRP ratio	7.23	84.4	71.1	0.812	0.001	0.722-0.902

## Discussion

This study suggests that the CA 19-9/CRP ratio may be more informative than CA 19-9 alone in determining malignancy in obstructive jaundice. This has clinical plausibility, as malignant obstruction is more prone to releasing tumour markers, and benign obstruction is sometimes accompanied by a stronger inflammatory response that can disproportionately raise CRP [18,19]. CA 19-9 is still commonly used in pancreaticobiliary malignancy; however, its interpretation alone has limited use. CA 19-9 can be increased by cholestasis and a variety of benign hepatobiliary conditions, thus lowering its sensitivity to malignancy [8,9]. CRP is similarly non-specific, as it rises in infection, tissue injury and cancer-related inflammation, but this characteristic may still be useful diagnostically because benign obstructive jaundice complicated by inflammatory biliary disease or cholangitis may show relatively greater CRP elevation than malignant obstruction [20,21].

In our study, the CA 19-9/CRP ratio had an AUC of 0.812, outperforming CA 19-9 alone and the CA 19-9/bilirubin ratio, suggesting better performance in differentiating malignant and benign cases and therefore better triage value in deciding who may need expedited malignancy-focused evaluation [11,18,21]. In comparison, the CA 19-9/bilirubin ratio had relatively worse performance. As bilirubin levels are more indicative of the presence of biliary obstruction and its severity, a significantly higher bilirubin level is not a hallmark of malignancy [9,19]. The prevailing clinical practice still directs that laboratory indicators are to be evaluated with clinical data and images and not as a determinant of malignancy on their own [1,2]. The variety of cases in the cohort also enhances the practicality of the results, as the performance of biomarkers depends on case mix and disease prevalence [11,16]. The CA 19-9/CRP ratio, however, cannot be regarded as an alternative to imaging, endoscopy or histopathology. MRCP, ERCP and tissue diagnosis are still necessary to establish anatomy, the cause of obstruction and the final pathology [5,22]. In general, the ratio can be considered an expedient complement to risk stratification in a larger diagnostic framework, and additional validation is required concerning various malignancy types and inflammatory conditions [4,21].

In the present study, malignant obstruction was associated with markedly increased CA 19-9 levels (median 370 U/mL) and a substantially higher CA 19-9/CRP ratio (median 42.92) compared with benign obstruction, while CRP showed the opposite pattern (Table 4). Similar observations were reported by Sayed et al., who demonstrated that the CA 19-9/CRP ratio improved discrimination between malignant and benign obstructive jaundice compared with CA 19-9 alone, although variations in cut-off values were observed between populations [24]. Zhou et al. also highlighted that the diagnostic value of CA 19-9 varies according to cancer type and clinical context, emphasising the need for combined approaches rather than isolated marker interpretation [25]. Lyu et al. suggested that combining CA 19-9 with other clinical indicators may improve prognostic interpretation in pancreatic malignancy [26]. Consistent with these reports, our ROC analysis demonstrated that the CA 19-9/CRP ratio achieved the highest discriminatory ability among the evaluated markers, with an AUC of 0.812, a sensitivity of 84.4% and a specificity of 71.1%. However, differences in inflammatory status, disease spectrum and diagnostic reference standards may explain variations in reported performance across studies. Previous biomarker studies have similarly emphasised that inflammatory markers and tumour markers should be interpreted together with imaging and pathological findings rather than used as independent diagnostic replacements [24,27,28].

There are several limitations in this study. This single-centre study included only 90 patients. Its limited sample size, referral-centre case mix and local management pathways may not represent patients attending other hospitals or populations. Purposive sampling may have introduced selection bias and limited representativeness. We explicitly acknowledge that the timing of blood collection relative to index-admission procedures and detailed processing/calibration/QC records could not be verified. These points have been listed for confirmation with the investigators and original laboratory records. In addition, approximately 5%-10% of individuals may produce little or no CA 19-9 because of their Lewis antigen phenotype; therefore, CA 19-9-based ratios may be unreliable in this subgroup. We also acknowledge that demographic imbalance and potential confounding were sources of bias, as no adjusted analysis is reported. The threshold of 7.23 was selected and assessed in the same derivation sample, without internal resampling or independent external validation. Consequently, the reported sensitivity and specificity may be optimistic, and the cut-off should be regarded as provisional until prospectively or temporally validated in a separate cohort. Larger, prospective, multicentre studies with prespecified cut-offs and external validation are needed before clinical implementation.

## Conclusions

In this selected, single-centre cohort, the CA 19-9/CRP ratio showed a higher observed AUC for distinguishing malignant from benign obstructive jaundice than CA 19-9 alone. The threshold of 7.23 yielded an apparent sensitivity of 84.4% and a specificity of 71.1%, but it was derived and assessed in the same sample and has not been independently validated. The CA 19-9/CRP ratio should be used as an exploratory adjunct requiring independent validation rather than as a clinically validated diagnostic threshold. Larger independent studies are required to evaluate reproducibility, clinical utility and an appropriate threshold before routine use is considered.
